# An Elementary Treatise on Human Anatomy

**Published:** 1861-03

**Authors:** 


### BOOK AND PAMPHLET NOTICES.

An Elementary Treatise on Human Anatomy. By JOSEPH LEIDY, M. D.

The above work, from the press of the well known firm of J. B. Lippencott & Co., Philadelphia, was announced in a former number of the Examiner. The book is worthy of more than a passing notice, and we desire briefly, again, to call the attention of the profession to its merits. Professor Leidy has given us a work concise, lucid and complete, so far as the wants of the student are concerned—one which we take peculiar pleasure in commending to our countrymen as comparing favorably with the best works in our own or any other language. An American work, with us, should have preference, if our national vanity is not gratified at the disparagement of better books, and we are happy to say that in several respects, we consider it superior to any elementary work which has yet appeared. None so much as a teacher realizes the necessity of a correct and uniform standard of pronunciation of anatomical names; though every educated man in our profession has noticed a sad discrepancy or deficiency in this respect. The work before us adopts an accentuation of technical terms, which, so far as we have noticed, is faultless, and of great assistance to a critical student.

We object to its *Anglicism* of the original, long used names of anatomical structures, which passing from the Latin and Greek to the modern languages, are recognized by all educated men alike. We believe that every medical student should attain such a knowledge of those languages, as would render the meaning of their terms as evident as though expressed in his own vernacular tongue.

The objection is in a measure obviated by the retention of the original names in foot notes, but these will be treated by the student very much as the author anticipated—the association incorporating in the mind the name italicised in the text.

The histological descriptions of the tissues are fine, and the figurings of microscopic appearances, *beautiful*, excelling any thing of the kind which we have met in any practical treatise on anatomy.

The work is illustrated by 392 engravings, well executed.— Those representing the nervous distribution are particularly fine.

In typographical appearance and mechanical execution, the Publishers have evidenced a degree of taste which leaves nothing further to be desired.

We most cordially commend it to the profession as not only meeting the wants of the student, but all ordinary requirements of the general practitioner. J. H. H.

